# The Influence of Polymer Impregnating Materials on Structural Strengthening of 3D Printed Sand Molds Produced by Binder Jetting Method

**DOI:** 10.3390/polym16212978

**Published:** 2024-10-24

**Authors:** Andrzej Rybak, Radek Javora, Robert Sekula, Grzegorz Kmita

**Affiliations:** 1 ABB Corporate Technology Center, Starowislna 13A, 31-038 Krakow, Poland; 2 ABB s.r.o, Videnska 117, 619 00 Brno, Czech Republic; radek.javora@cz.abb.com; 3 Hitachi Energy Research, Pawia 7, 31-154 Krakow, Poland; robert.sekula@hitachienergy.com (R.S.); grzegorz.kmita@hitachienergy.com (G.K.)

**Keywords:** polymer impregnation, additive manufacturing, sand mold, structural strengthening

## Abstract

Additive manufacturing offers great potential for various industrial solutions; in particular, the binder jetting method enables the production of components from various materials, including sand molds for casting. This work presents the results of an extensive set of experiments aimed at enhancing the structural strengthening of 3D-printed sand molds. Structural strengthening was achieved by impregnating the sand-printed structures with two polymer materials: epoxy resin and silicone varnish. Impregnation was performed with variable parameters, such as temperature, pressure, and time. Structural strengthening using polymers was investigated by analyzing the flexural strength and impact resistance of the impregnated products and comparing these obtained values with the reference material in terms of impregnation parameters and the polymer used. Microstructural observations and an analysis of the pore filling were also performed. This approach allowed for a full assessment of the influence of processing parameters and the type of polymer used for impregnation on the properties of sand-printed structures, which allowed for identifying the most optimal method to be used to strengthen the sand molds for casting the components for electrical devices. As a direct proof of concept, it was shown that impregnation with polymeric materials could effectively strengthen the sand mold, increasing its flexural strength and impact resistance by over 20 times and 5 times, respectively. A full-scale mold was printed using binder jetting, impregnated with epoxy resin at 65 °C, and used to successfully fabricate a fully functional electrification device.

## 1. Introduction

The technological development of the present world is currently striving to develop manufacturing processes, materials, and products that are friendly to our planet; for many companies, sustainability is a key pillar of their strategy [1,2]. This means that the industry is guided by the idea of producing parts and devices that can be reused, recycled, or naturally decomposed in the environment. Additive manufacturing has great potential for this type of solution and is considered one of the fastest-growing areas in the last decade [3,4]. 3D printing, when initially developed in the 1990s, was considered a technology suitable only for prototype production. However, it is now increasingly considered an industrial production technology due to its high precision, product repeatability, and wide range of materials. Additionally, 3D-printed objects can have very complex shapes and can provide a number of extra functionalities that cannot be achieved using any traditional manufacturing methods [5,6].

The methods that have attracted great interest include, among others, the binder jetting method, which enables the production of components from various materials, such as sand, gypsum, ceramics, and metals [7]. Among these methods, binder jetting of sand seems to be one of the innovative 3D printing solutions with numerous commercial applications [8]. This process is very economical because sand is a readily available material that can be recycled, and its use in industry can contribute to the targeted development of more ecological products.

The 3D binder jetting printing method was developed in the early 1990s by scientists from the Massachusetts Institute of Technology (MIT). It consisted of selectively bonding gypsum powder with a binder that was sprayed through the head. Objects were printed on a powder bed, gradually creating parts of a given object. In 1999, the German start-up Generis invented a similar method to MIT, but instead of gypsum powder, sand was used. Generis is also the first company to produce a 3D printer for producing sand casting molds. Over the years, Generis has transformed into ExOne, simultaneously becoming one of the largest providers of this type of service in the world. There are currently several companies that deal with 3D printing with sand. The main market leaders are Voxeljet and the aforementioned ExOne [9,10,11], recently acquired by Desktop Metal.

In the 3D binder jetting printing process, a binder is fed through the head to the powder substrate in very small amounts in the form of drops with a diameter of about 80 μm. The application of the droplets causes the formation of spherical agglomerates consisting of binder and powder particles, with such agglomerates also bonding to the previously printed layer. After producing one layer of printing, the bed is lowered downwards, and then a new layer of powder is applied in its place. This cycle is repeated until the printed part reaches its original dimensions. The powder substrate is bonded together thanks to the head, which supplies the binder in small amounts, thus causing the powder grains to stick together and produce the printed specimens [9]. The process of creating printed specimens in a powder bed has many advantages, such as speed of printing, with little binder escaping through the head, and no need for support elements for complex designs because the sand bed is self-supporting. Another advantage is the ability to design elements in one plane, which allows for an increase in the number of parts produced during a single printing cycle. Thus, the rate of binder deposition is high, and the total cost of printing with binder jetting is low due to the lack of need for a high-power energy source [9].

However, the main disadvantages of binder jetting products are their relatively low accuracy and rough surface finish, as well as, in cases such as sand printing, low mechanical strength and integrity, which makes it necessary to use an additional infiltration process that will improve the mechanical properties of the printed specimens [9,11]. Infiltrants, i.e., liquid substances, usually based on resins, e.g., acrylic, are used to strengthen 3D parts made by binder jetting, resulting in greater grain cohesion and higher mechanical strength.

There are reports that products made of polymer powders can be used as casting molds, and metal powders enable the printing of machine parts or prototypes. On the other hand, there are also reports that elements made of silica-based powders can be used as casting molds in the automotive and heavy industries [9,11]. It is precisely in connection with the latter that a wide growth of metal casting using sand-printed molds is currently observed, taking into account the printing speed and relatively low cost of this technology [12,13,14]. An example of a popular application of sand molds is metal casting molds, the dimensions of which are impressive and can reach up to several meters in size. Of course, in such an application, the very high mechanical strength of the mold body is not required because after casting the metal and its solidification, the mold is disintegrated to obtain the final product. However, in the case of the need to use such sand-printed molds for multiple castings, e.g., in polymer molding, it is necessary to minimize the brittleness of the material and improve the mechanical properties and integrity. As already mentioned, improvement of this property can be achieved by the impregnation process, i.e., filling the pores (voids with air) with a substance that will increase the consistency of the product by connecting sand grains with each other [15].

This paper describes an innovative attempt to study the possibility of using sand printing technology to produce reusable molds for casting thermosetting materials commonly used as insulation in electrical devices in the energy industry. The paper presents the results of research conducted in a wide range of experiments aimed at improving structural reinforcement by impregnating sand molds with two exemplary infiltrators, namely epoxy resin and silicone varnish, which were selected based on preliminary studies as the most promising. The infiltrators mentioned were selected, taking into account the possibility of increasing mechanical integrity but also giving the potential for later separation of the casting from the mold with or without the use of a release agent (separator). The ability to improve the mechanical integrity of the sand molds of the tested infiltrants was checked under variable process parameters such as temperature, pressure, and time. The degree of structural strengthening was assessed by testing the flexural strength and impact resistance and comparing the obtained values with the reference material, which was an un-infiltrated sand print. Microstructural observations and a pore-filling analysis were also carried out. The conducted studies allowed for a full evaluation of the effect of processing parameters and infiltrating medium on the properties of sand-printed structures, which allowed for the identification of the most optimal type of solution to be used for strengthening sand molds used for casting electrical device components. As a direct proof of concept that impregnation with polymeric materials can effectively strengthen the sand mold, a full-size sand mold was impregnated with the tested polymeric materials and used to successfully fabricate an electrification device.

## 2. Materials and Methods

### 2.1. Epoxy Resin Formulation

The epoxy resin formulation CHS-EPODUR 494-1667 (Spolchemie, Usti nad Labem, Czech Republic) was used in the impregnation process. This system is used in the electrical and electronics industries [16,17,18,19] and is suitable for high- and medium-voltage applications [20,21,22,23]. Selected properties of the raw material are presented in Table 1.

The entire resin system is obtained by mixing the four components, namely epoxy resin, hardener, accelerator, and flexibilizer, in the appropriate proportions according to the scheme shown in Table 2.

Mixing the components A, B, C, and D in the right proportions guarantees a resin with the required properties. The substances should be thoroughly mixed with each other, applied to the intended place, and hardened. The resin cross-linking process is best carried out in an oven, starting from 5 h at 80 °C, then 3 h at 140 °C, and ending with 8 h at 20 °C. The resin hardened in this way is a solid and is characterized by mechanical robustness.

### 2.2. Silicone Varnish

SARSIL W silicone varnish produced by Silikony Polskie (Nowa Sarzyna, Poland) was used, the selected properties of which are presented in Table 3.

SARSIL W is intended for the strengthening and simultaneous hydrophobization of all types of absorbent, porous materials, e.g., fine and coarse pore sandstones, limestone, etc. It reduces water absorption and increases mechanical properties. The use of silicone varnish as an impregnation guarantees resistance to weather conditions, UV radiation, and many chemical factors. In order to obtain SARSIL W with the described properties, it is necessary to mix two components, SARSIL W and Hardener W, in a weight ratio of 100:4.

Hardener W is a catalyst for hydrophobic agents. Basic information on the Hardener W is included in Table 4.

### 2.3. Manufacturing of Printed Structures

**Figure 1:**
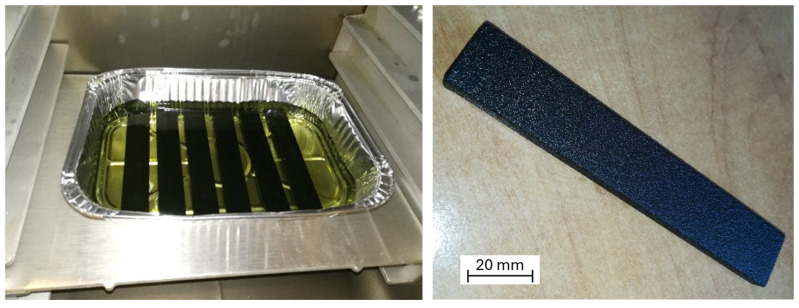
Samples during the impregnation process in a vacuum oven (**left**) and sample impregnated with epoxy resin (**right**).

The samples intended for impregnation in a bar shape with dimensions of 8 × 20 × 120 mm were produced using the ExOne (North Huntingdon, PA, USA) binder jetting 3D printing method [9,10,11], using quartz sand (SiO_2_) FS001 as a raw material, and a cold-curing phenolic binder FB201, with the printed layer thickness of 0.26 mm (see Figure 1).

In the printed specimens, the grains of sand are bonded together with a small amount of thermosetting resin, and the parts contain 99% quartz sand and 1% resin in their volume. The grain size of quartz sand varies from 0.05 to 2 mm. Due to the specific nature of the material used, consisting of quite large sand particles, the printed specimens have numerous pores in their structure. It is estimated that the pores occupy 30% of the total print volume. Sand grains in the samples have very low cohesion, and under the influence of slight mechanical friction, they detach from the surface of the material, causing weight loss and changes in dimensions. The samples are also characterized by a very low mechanical strength and high brittleness.

### 2.4. Impregnation Process

The process of impregnating the sand-printed specimens consists of introducing the impregnating substances into the pores of the material and thus removing air from the sample volume. The impregnation process was modified by four parameters, which were the following:

- type of impregnating substance,
- pressure during the process,
- temperature of impregnation,
- time of process.

The adjustment of the impregnation parameters is used to extract a set of parameters that give the best performance of impregnated parts. Two impregnating substances were used: epoxy resin and silicone varnish (see description in Section 2.1 and Section 2.2).

The samples were impregnated at room temperature (23 °C) or at a slightly elevated temperature of 65 °C. They were subjected to a vacuum of 5 mbar or an atmospheric pressure of 1000 mbar. They also varied in the impregnation time, which was 1 or 5 min, depending on the sample. The full matrix of the impregnation parameters for the produced samples is summarized in Table 5.

The process of impregnating the samples with epoxy resin required a change in the temperature parameter due to its high viscosity at room temperature. The resin at 65 °C has a lower viscosity, which makes it easier to flow into the pores of the printed structure. In the case of silicone varnish as an impregnation medium, increasing the temperature was not necessary as these substances are intrinsically low in viscosity.

The impregnation pressure during the process was modified, as it was assumed that the reduction of the pressure in the process should lead to removing air from the pores, thereby facilitating the filling of the pores with the impregnating substance. The modification of the impregnation time was aimed at determining the influence of the length of the immersion time of the samples in the impregnation medium on their physical and thermomechanical properties. To verify the repeatability of the results, six samples were made for each characteristic parameter setting. An example of the impregnated samples is shown in Figure 1.

### 2.5. Microstructure of Impregnated Structures

The scanning electron microscope (SEM) enables the characterization of the surface structure of the investigated samples and chemical composition. The micrographs were taken using the Jeol JSM-5510LV (Tokyo, Japan) microscope in order to compare the morphology of the impregnated samples with the reference sample. Before SEM observation, the structures were sputtered with a 10 nm layer of gold.

### 2.6. The Degree of Pore Filling by Infiltrants

Density is a physical property of bodies that determines the dependence of body weight on its volume, according to the following formula:

(1) $$\rho =\frac{m}{V}\left(\frac{kg}{m^{3}}\right)$$

where *m* is mass of the substance in kg, and *V* is a sample volume in m^3^.

The measurement is carried out using a hydrostatic balance, which measures the density of the body based on the Archimedes law [24]. The measurement consists of measuring the mass of the substance twice: in the air and in the standard liquid (ethyl alcohol 96% was used in the test). The values displayed by the balance are smaller in the latter case since the mass of the substance is reduced by the buoyancy of the standard liquid used. The ratio of the body mass in the air to the buoyancy force allows the determination of the dimensionless quantity A; it defines the ratio of the density of the tested body ρ to the density of the standard liquid ρ_c_ in which the body is immersed [24]:

(2) $$A=\frac{m_{air}}{F_{w}}=\frac{\rho }{\rho_{c}},$$

where *m_air_* is a body mass in the air (kg), *F_w_*—buoyancy force of the body by the standard liquid (N), *ρ*—density of the tested body (kg/m^3^), *ρ_c_*—density of the reference liquid (kg/m^3^), and *A*—dimensionless quantity.

The buoyancy force is equal to the difference between the weight of the body in the air and in the standard liquid. Hence, the density of the test substance is determined according to the following formula [24]:

(3) $$\rho =A\cdot \rho_{c}.$$

When testing the density of highly porous substances with the hydrostatic method, it is important to remember the fact that the standard liquid flows between the pores, thus influencing the correct density result. The higher the calculated density, the greater the number of pores in the material. The percentage of pore filling by the impregnating substance was calculated by comparing the density of the reference sample to the density of the impregnated samples.

### 2.7. Mechanical Tests

Flexural properties are the most commonly investigated mechanical properties of the materials. Samples in the form of rectangular bars were measured using a Universal Testing System 3367 (Instron, Norwood, MA, USA) in order to perform the three-point flexural test according to ISO 178 standard [25].

Impact resistance determines the material’s resistance to cracking under dynamic loading according to ISO 13586 [26]. The impact resistance of printed sand samples was determined using a Charpy hammer for samples of the same dimensions with a notch depth of 1 mm. It is determined by the ratio of the impact energy needed to break the material to the cross-sectional area of the sample where the notch is located, according to the following formula:

(4) $$KC=\frac{K}{S}\left(\frac{J}{{cm}^{2}}\right),$$

where *KC* is an impact resistance, *K* is impact work (J), and *S* is cross-section area at notch location (cm^2^).

## 3. Results and Discussion

### 3.1. Analysis of Impregnated Structures

The selected samples from Table 5 were observed in order to compare them with the untreated reference. The images below show the micrographs of investigated samples taken with SEM at 100 times magnification.

**Figure 2:**
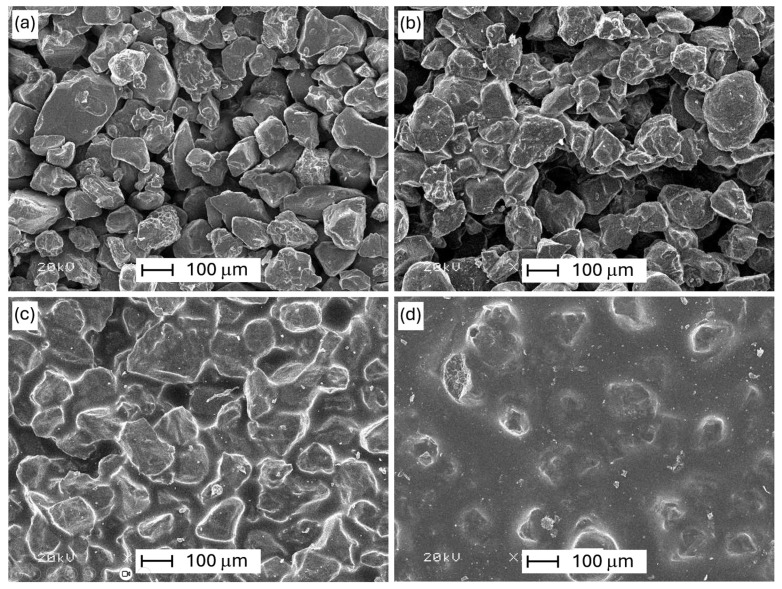
SEM images: (**a**) reference sample, and samples impregnated with (**b**) silicone varnish (RT, 5 mbar, 5 min); (**c**) epoxy resin (65 °C, 1000 mbar, 5 min); (**d**) epoxy resin (65 °C, 5 mbar, 5 min).

Scanning electron micrographs show how the morphology of the impregnated samples differs compared to the reference sample. Figure 2a shows the morphology of the reference sample, which is characterized by a granular structure with a highly porous structure. The grain size range is 50–400 μm, and the structure appearance is not uniform.

The sample impregnated with silicone varnish (cf. Figure 2b) has a morphology in which the filling of voids is hardly noticeable and contains a lot of unfilled pores. It can be seen that there are small clusters of grains that are connected with each other by the impregnation medium; in these places, inter-grain connections in the form of “bridges” are created. The pore-filling efficiency is low, the appearance of the sample is very similar to the reference, and the structure is heterogeneous.

On the other hand, the sample impregnated with the epoxy resin using the parameters 65 °C, 1000 mbar, and 5 min has a morphology (see Figure 2c) in which it is noticeable that the voids between the grains are filled very thoroughly by the impregnating polymer material. The filling efficiency is high, and there are no voids without the resin. In the impregnated surface, outlines of grain shapes are visible, which are conformally covered with a layer of impregnating medium.

In the case of the next sample (see Figure 2d), which was impregnated with the resin at the same temperature and time as the previous one (65 °C, 5 min) but in a vacuum (5 mbar), it can be seen that filling the voids between the grains with the impregnating substance is very precise. The filling efficiency is very high, and no voids were noticed, while the impregnated surface shows a small amount of not-very-clear grain outlines.

As a result of the SEM micrographs analysis, it can be concluded that using the resin as an impregnating substance at reduced pressure (5 mbar), increased temperature of 65 °C, and longer time (5 min), the most accurate filling of pores and smoothing of the surface structure was obtained, as shown in Figure 2d. The resin sample impregnated with epoxy resin at 1000 mbar is characterized by slightly worse filling. On the other hand, structures containing silicone varnish are impregnated to a much lesser extent.

### 3.2. Pore Filling Determined by the Hydrostatic Method

Calculations of the percentage of pore filling by the active substance were made by comparing the value of the reference sample density to the density of the impregnated samples (see Section 2.6). The values of the average percentage of pore filling by the impregnating medium for all tested samples are presented in Table 6.

Samples impregnated with epoxy resin are characterized by the highest percentages of pore filling in the range of 17–24%. This group includes samples impregnated under atmospheric pressure (1000 mbar), and they have the highest pore-filling values (about 21–24%). The structures obtained under the influence of the reduced pressure (5 mbar) are characterized by a lower degree of filling (approx. 17%). The distribution of the values obtained during the tests may have been disturbed because, during the atmospheric impregnation, the resin fills the pores located only on the outer parts of the sample and sticks to it only on the surface, building a tight layer. With these types of samples, the liquid cannot fill the pores during hydrostatic testing due to the thick layer of resin on the outer parts of the structures. For this reason, the percentage of pore filling in samples impregnated at atmospheric pressure may be higher than those impregnated at 5 mbar without showing the actual state of filling. One can see that the temperature of the used resin and the impregnation time do not have a significant effect on the degree of pore filling.

Samples impregnated with silicone varnish are characterized by the lowest values of the percentage of pore filling in the range of 2–12%. In this group, the samples impregnated under a pressure of 5 mbar can be distinguished because they have the highest values of pore filling (about 12%). A worse degree of filling (about 2–8%) is characteristic of structures obtained under the influence of atmospheric pressure. No silicone build-up was observed on the outer portions of the sample during the atmospheric pressure impregnation process; consequently, the results of percentage pore filling are not affected. The reduced pressure has a positive effect on the degree of filling the samples with the impregnating medium. Atmospheric pressure samples have lower pore-filling values because the impregnating substance is able to penetrate only a small part of the pores without the effect of low pressure. The time of the impregnation process only affects the samples produced at atmospheric pressure; a longer residence time of the samples in the medium results in better filling of the structures (by about 6%).

When analyzing the percentage degree of pore filling, it was noticed that the optimal results were obtained only for silicone-impregnated samples. This experience showed that epoxy resin is too viscous a substance, which, without the influence of reduced pressure (5 mbar), causes ineffective impregnation, and the substance deposits only on the surface of the samples, forming a kind of “shell”. Silicone varnish is distinguished by its properties: it has a low viscosity and good infiltration properties that allow the medium to flow between the pores without creating an impermeable layer.

### 3.3. Mechanical Properties

The purpose of modifying the printed sand structures was to improve their mechanical properties and integrity by introducing the impregnation polymer materials (epoxy resin and silicone varnish) into the voids existing in the print. The effectiveness of the impregnation was evaluated by examining the mechanical properties, such as flexural strength and impact resistance, and then comparing the results with the properties of a reference material.

**Figure 3:**
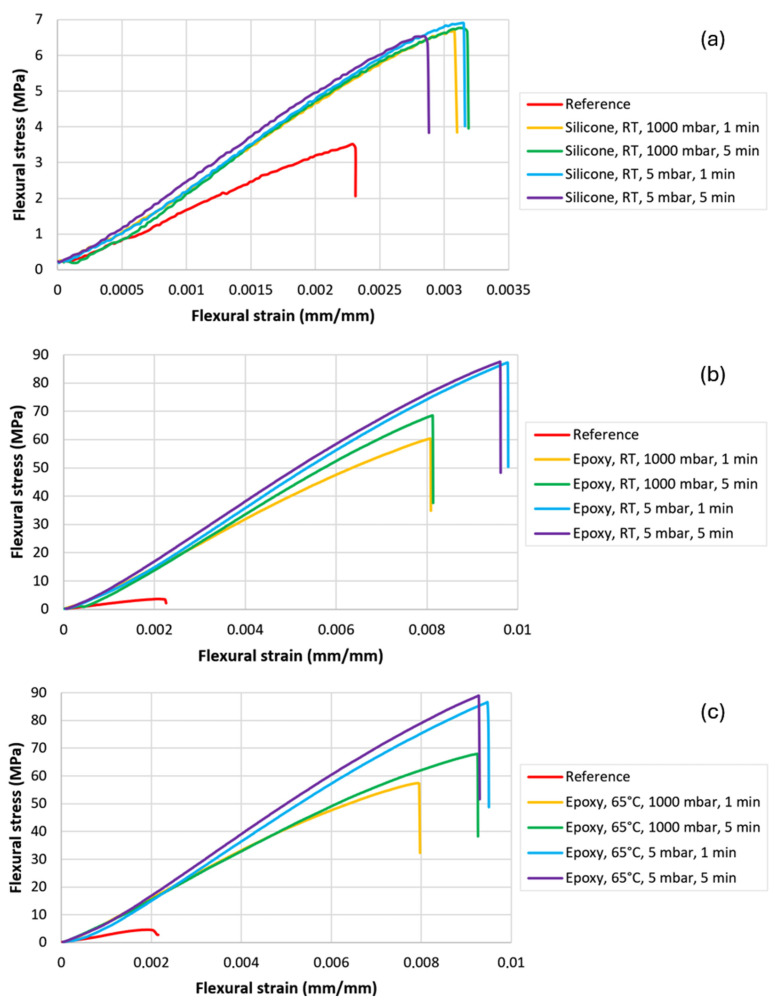
Representative stress–strain relationships during flexural test of samples impregnated with (**a**) silicone varnish, (**b**) epoxy resin at room temperature, (**c**) epoxy resin at 65 °C.

Figure 3a–c illustrate the dependence of flexural stress on material deformation for samples impregnated with various polymers in comparison with a reference sample (not impregnated).

After analyzing the following graphs, the characteristic values can be evaluated, such as maximum deformation and maximum flexural stress at break. The determined average values for all tested samples and their standard deviations are presented in Table 7.

The flexural strength for reference non-impregnated samples did not exceed 4 MPa. The highest values of the maximum stress at break were recorded for samples impregnated with epoxy resin (56–88 MPa), with a significant influence of the impregnation conditions on the maximum strength values. The highest values of flexural strength (about 87–88 MPa) were obtained for samples impregnated under the low pressure of 5 mbar, while samples impregnated under atmospheric pressure had a strength of 56–68 MPa, which depended quite strongly on the impregnation time. The temperature of the applied epoxy resin had no significant effect on the mechanical properties.

Samples impregnated with silicone varnish reached maximum stress values of 6.5–6.8 MPa, which, compared to the reference sample, indicates a relatively significant increase; although, from the point of view of functional properties, this means only a slight improvement in properties after impregnation. For silicone varnish-impregnated samples, only a slight influence of the impregnation conditions on the final properties was observed.

The highest strain at break was recorded for the sample impregnated with epoxy resin, with a value of 0.75–0.97%, which is many times higher when compared to the reference sample (0.23%). As with the flexural strength results, the strain at break values was found to be the highest in the samples impregnated at 5 mbar (approximately 0.9%), while those impregnated at atmospheric pressure had lower strain at break values (ca. 0.8%). Thus, it can be stated that lowering the impregnation pressure below atmospheric pressure has a beneficial effect on the properties of the samples. The time of impregnation and the temperature of the applied epoxy resin in the case of most samples did not significantly affect their properties. Samples impregnated with silicone varnish were characterized by low values of strain at break at the level of 0.29–0.32%, which indicates only a slight improvement in properties compared to the reference. The time and pressure of impregnation did not significantly affect the strain values for both tested impregnation materials.

The average impact resistance values and their standard deviations measured for all tested samples are presented in Table 8. The highest impact resistance was noted for samples impregnated with epoxy resin, reaching values in the range of 0.32–0.55 J/cm^2^. Comparing the properties of the printed and impregnated structures of this group with the reference sample (0.109 J/cm^2^), one can notice a significant improvement in properties. The samples impregnated under a pressure of 5 mbar can be distinguished as having the highest impact resistance values (about 0.5 J/cm^2^), while samples impregnated with resin under atmospheric pressure have slightly worse properties. The temperature of the resin applied during impregnation has a beneficial effect on the impact resistance values only in the case of samples impregnated under atmospheric pressure, while samples prepared under 5 mbar did not show such dependencies, and the temperature did not significantly affect the impact resistance. Furthermore, the impregnation time for most samples did not significantly affect the impact resistance of sand structures.

Samples impregnated with silicone varnish were generally characterized by the lowest values of impact resistance, in the range of 0.127–0.131 J/cm^2^, and only a very slight improvement in properties can be noted here (by about 0.02 J/cm^2^). No significant influence of pressure and impregnation time on the impact resistance values of the samples was observed.

### 3.4. Full-Scale Proof of Concept

**Figure 4:**
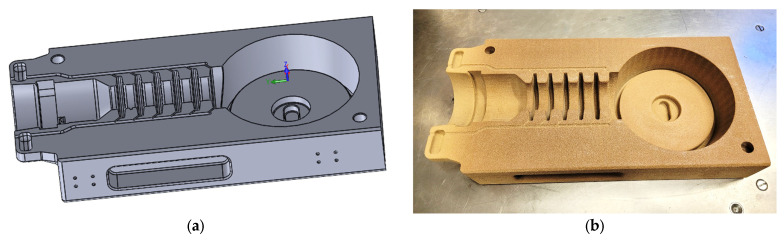
(**a**) CAD model, and (**b**) real-size 3D printed half of sand mold after impregnation with epoxy resin.

To perform the proof of concept, a full-size sand mold for the vacuum casting of a current transformer with resin insulation was 3D printed according to the CAD model shown in Figure 4, with the outer dimensions of one-half of the mold being 450 × 220 × 85 mm and weighing 7.1 kg. The mold was printed using ExOne printing technology using FS001 quartz sand and FB201 cold-cured phenolic binder, with a printed layer thickness of 0.26 mm [9,10,11,27].

After printing, the mold was impregnated with epoxy resin in a vacuum chamber at room temperature and 5 mbar (see Section 2.4). Due to the large dimensions of the mold, the impregnation time was significantly longer compared to the small test samples, and the mold was kept completely immersed in epoxy resin until the release of air bubbles stopped visibly (approx. 25 min for each half of the mold). After impregnation, the mold was placed in an oven to harden the epoxy resin, which penetrated the printed structure.

In the next step, a release agent was applied to the mold surface to prepare the mold for casting the resin-insulated electrical device. The additional release agent treatment was considered essential to avoid the adhesion of the cast device to the mold since the aim of the study was to reuse the mold multiple times and perform multiple casting processes using one mold.

**Figure 5:**
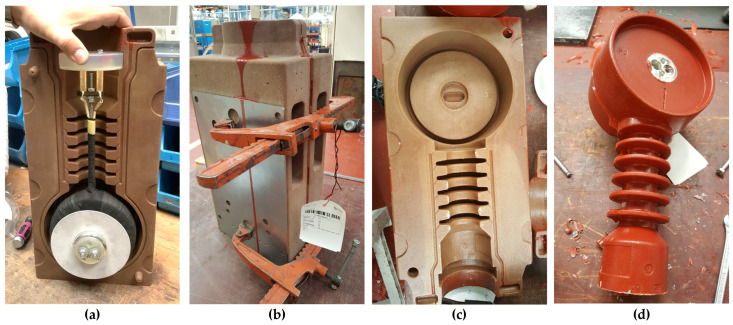
Images showing the steps involved in producing an electrification device using a sand mold: (**a**) half of epoxy-impregnated sand mold with assembled internal parts of current transformer; (**b**) two halves of mold closed and secured with clamps after filling with casting composite in vacuum oven; (**c**) sand mold after demolding of the casted device; (**d**) de-molded current transformer.

The prepared mold was used to fabricate an exemplary electrical device, namely an indoor current transformer, as shown in Figure 5. First, the internal components of the device were assembled inside the mold, as illustrated in Figure 5a. Then, both halves of the mold were closed and secured with clamps (see Figure 5b) in order to avoid opening during the casting process. In the next step, the mold was placed into the vacuum oven, filled with pre-mixed epoxy resin with hardener and silica flour, and cured according to the heating profile recommended by the resin manufacturer.

After curing, the mold was opened (see Figure 5c) and the cast device was successfully de-molded (see Figure 5d). No cracks or damage to the sand mold were observed, and the de-molded device was of good quality with no surface defects. In order to evaluate the long-term effect of the polymer impregnation and its influence on the durability and robustness of sand molds, the device casting process was repeated 10 times without visible influence on the quality of both the mold and the fabricated device. Additionally, all the current transformers fabricated with the use of the polymer-strengthened sand mold successfully passed all the required functional tests, which finally proved the applicability of the proposed technical solution. The obtained results are very important from an application point of view, especially in the case of rapid prototyping or production of short series using 3D-printed tools for casting electrification devices.

Additionally, it is worth noting that the cost of the sand-printed mold was 125 times lower than standard aluminum machined mold, and the delivery time was shortened from six weeks to only one week, which has a critical influence, especially in cases of a need for rapid prototyping of new designs of electrical apparatuses.

## 4. Conclusions

It has been shown that the crucial parameters required from an application point of view can be improved, and in particular, the following conclusions can be drawn:

- Micrographic analysis of the impregnated structures indicated that the samples impregnated with epoxy resin are characterized by the highest pore filling, whereas impregnation with silicone varnish was less effective.
- Based on the test results of flexural strength and strain at break, it can be concluded that the samples impregnated with epoxy resin have the best properties, exceeding the properties of the reference samples more than twenty times, whereas the properties of the samples impregnated with silicone varnish exhibit only a two-fold increase.
- The impact resistance results also confirmed that the samples impregnated with epoxy resin possess the highest performance with a five-fold enhancement in relation to the reference samples. In comparison, the samples impregnated with silicone varnish show only a 20–30% increase in the impact resistance.
- A direct proof of concept was performed, which confirmed that polymer impregnation can effectively strengthen a full-scale sand mold.
- The durability of the impregnated sand mold was successfully verified as the mold was used 10 times for the industrial-scale fabrication of the electrification devices.

The performed evaluation of the methods for structural strengthening of the printed sand molds using polymer impregnation has clearly indicated that the functional parameters of sand molds can be fully optimized to successfully cast the components for electrical devices.

## 5. Patents

Robert Sekula, Grzegorz Kmita, Andrzej Rybak, Dariusz Bednarowski, Lukasz Matysiak, Radek Javora. 2022. Method of Preparation of Sand Casting Molds with A Protective Coating. EP3802042B1, July 6.

## Figures and Tables

**Table 1:** Characteristic properties of epoxy resin formulation.

Properties	Description or Value	Unit
Glass transition temperature T_g_	100–115	°C
Viscosity (@ 45 °C)	25,000	mPa·s
Pot-life (@ 60 °C)	8	h
Flexural strength	120–150	MPa
Tensile strength	75–90	MPa
Elongation	5–10	%
Impact resistance	20	kJ/m^2^

**Table 2:** Weight proportions of epoxy resin components.

Ingredient Designation	Trade Name	Function	Proportion by Weight
A	CHS-EPODUR 494-1667 comp. A	Epoxy resin	100
B	CHS-EPODUR 494-1667 comp. B	Hardener	85
C	CHS-EPODUR 494-1667 comp. C	Accelerator	0.6
D	CHS-EPODUR 494-1667 comp. D	Flexibilizer	20

**Table 3:** Characteristic properties of SARSIL W silicone varnish.

Properties	Description or Value	Unit
Density (@ 20 °C)	0.82	g/cm^3^
Viscosity (Ford cup @ 20 °C)	11	s
Temperature of solidification	<−20	°C
Boiling range	150–205	°C
Flash-point	47	°C
Temperature of self-ignition	>230	°C

**Table 4:** The characteristic properties of Hardener W.

Properties	Description or Value	Unit
Density	0.81–0.83	g/cm^3^
Viscosity (Ford cup @ 20 °C)	25	s
Boiling temperature	150	°C
Flash-point	30	°C
Temperature of self-ignition	>230	°C

**Table 5:** Impregnation parameters of all produced samples.

Impregnating Substance	Temperature (°C)	Pressure (mbar)	Impregnation Time (min)
Epoxy resin	23	5	1
	23	5	5
	65	5	1
	65	5	5
	23	1000	1
	23	1000	5
	65	1000	1
	65	1000	5
Silicone varnish	23	5	1
	23	5	5
	23	1000	1
	23	1000	5

**Table 6:** Pore filling degree by investigated infiltrates.

Sample Type	Pore Filling Degree (%)
Reference	0
Silicone, RT, 1000 mbar, 1 min	2.31
Silicone, RT, 1000 mbar, 5 min	8.41
Silicone, RT, 5 mbar, 1 min	11.95
Silicone, RT, 5 mbar, 5 min	11.94
Epoxy, RT, 1000 mbar, 1 min	21.41
Epoxy, RT, 1000 mbar, 5 min	23.76
Epoxy, RT, 5 mbar, 1 min	17.88
Epoxy, RT, 5 mbar, 5 min	17.77
Epoxy, 65 °C, 1000 mbar, 1 min	22.04
Epoxy, 65 °C, 1000 mbar, 5 min	22.89
Epoxy, 65 °C, 5 mbar, 1 min	17.55
Epoxy, 65 °C, 5 mbar, 5 min	17.73

**Table 7:** Flexural mechanical properties of the tested samples.

Sample Type	Flexural Strain at Break		Flexural Strength at Break	
	(%)	Std. Dev.	(MPa)	Std. Dev.
Reference	0.229	0.006	3.39	0.158
Silicone, RT, 1000 mbar, 1 min	0.306	0.002	6.49	0.180
Silicone, RT, 1000 mbar, 5 min	0.301	0.013	6.59	0.170
Silicone, RT, 5 mbar, 1 min	0.315	0.002	6.82	0.085
Silicone, RT, 5 mbar, 5 min	0.29	0.010	6.46	0.070
Epoxy, RT, 1000 mbar, 1 min	0.749	0.076	56.38	6.481
Epoxy, RT, 1000 mbar, 5 min	0.802	0.112	63.77	9.659
Epoxy, RT, 5 mbar, 1 min	0.971	0.016	88.18	2.344
Epoxy, RT, 5 mbar, 5 min	0.949	0.017	87.40	1.392
Epoxy, 65 °C, 1000 mbar, 1 min	0.867	0.054	57.40	2.369
Epoxy, 65 °C, 1000 mbar, 5 min	0.975	0.035	67.93	0.229
Epoxy, 65 °C, 5 mbar, 1 min	0.905	0.029	86.32	1.605
Epoxy, 65 °C, 5 mbar, 5 min	0.928	0.006	87.84	2.677

**Table 8:** Impact resistance of the tested samples.

Sample Type	Charpy Impact Resistance ^1^ (J/cm^2^)	Std. Dev.
Reference	0.109	0.002
Silicone, RT, 1000 mbar, 1 min	0.129	0.005
Silicone, RT, 1000 mbar, 5 min	0.127	0.003
Silicone, RT, 5 mbar, 1 min	0.129	0.007
Silicone, RT, 5 mbar, 5 min	0.131	0.002
Epoxy, RT, 1000 mbar, 1 min	0.324	0.085
Epoxy, RT, 1000 mbar, 5 min	0.384	0.156
Epoxy, RT, 5 mbar, 1 min	0.551	0.053
Epoxy, RT, 5 mbar, 5 min	0.526	0.146
Epoxy, 65 °C, 1000 mbar, 1 min	0.509	0.011
Epoxy, 65 °C, 1000 mbar, 5 min	0.462	0.137
Epoxy, 65 °C, 5 mbar, 1 min	0.476	0.128
Epoxy, 65 °C, 5 mbar, 5 min	0.517	0.078

^1^ Cross-section at notch was 1.52 cm^2^ for all samples.

## Data Availability

The original contributions presented in the study are included in the article, further inquiries can be directed to the corresponding author.
